# Impact of serum eicosapentaenoic acid/arachidonic acid ratio on overall survival in lung cancer patients treated with pembrolizumab: a pilot study

**DOI:** 10.1038/s41598-024-51967-y

**Published:** 2024-01-16

**Authors:** Ikue Tanaka, Yukihiro Yano, Masahide Mori, Satoru Manabe, Keisuke Fukuo

**Affiliations:** 1 Department of Food Sciences and Nutrition Major, Graduate School of Human Environmental Science, Mukogawa Women’s University, Nishinomiya, Japan; 2 Department of Thoracic Oncology, National Hospital Organization, NHO Osaka Toneyama Medical Center, Toyonaka, Japan; 3 Research Institute for Nutrition Sciences, Mukogawa Women’s University, Nishinomiya, Japan; 4 Department of Nutrition, National Hospital Organization, NHO Osaka Toneyama Medical Center, Toyonaka, Japan

**Keywords:** Medical research, Oncology

## Abstract

This pilot study analyzed the dietary patterns of patients with non-small cell lung cancer undergoing initial pembrolizumab, an immune checkpoint inhibitor (ICI), treatment in the month before treatment. Serum fatty acid fractions and their associations with ICI treatment efficacy were also investigated. The results showed that long-term survivors (those who survived for ≥ 3 years) consumed significantly more seafood than short-term survivors (those who survived for < 3 years). Furthermore, the serum levels of eicosapentaenoic acid (EPA) as well as the ratio of EPA to arachidonic acid (EPA/AA) were higher in the long-term survivors than those in the short-term survivors. The group with a high serum EPA/AA ratio had a significantly higher overall survival rate after ICI treatment than the group with a low serum EPA/AA ratio. In conclusion, higher dietary seafood consumption may improve OS in lung cancer patients treated with ICI and the serum EPA/AA ratio may be a useful biomarker for determining the efficacy of ICI treatment. Thus, supplements that increase the serum EPA/AA ratio could serve as new nutritional interventions for enhancing the efficacy of ICI treatment. However, further large-scale case and intervention studies are required.

## Introduction

Immune checkpoint inhibitors (ICI) exert their anticancer effects by inhibiting the action of immune checkpoint molecules, such as programmed cell death 1 ligand (PD-L1), and activating the antitumor activity of CD8^+^ T lymphocytes^1^. ICI provide significant and sustained therapeutic effects in patients with stage IV malignant melanoma and non-small cell lung cancer (NSCLC)^2,3^. However, the 5-year follow-up results of a phase III study (KEYNOTE024) showed that the response rate of ICI was 46.1% and that more than half of the patients were ineligible^4^. Clarification of the factors that improve therapeutic efficacy is an urgent issue. The mechanism of action of ICI suggests that patients with high PD-L1 expression in tumor tissues respond well to ICI therapy. However, in some cases, the expression level of PD-L1 in the collected tumor tissue does not necessarily correlate with the therapeutic effect of ICI^5^.

A growing body of evidence is accumulating to suggest that dietary interventions can modulate nutrient availability in the tumor microenvironment (TME) and may enhance T-cell killing activity in cancer therapy^6,7^. For example, a ketogenic diet was reported to enhance tumor-reactive immune responses in the TME through a significant reduction in the expression of the immune check points CTLA-4 and PD-1 on glioma cells in a mouse model of malignant glioma^8^. Rubio- Patiño et al. reported that a reduction in dietary protein intake without an overall change in caloric intake may induce anticancer immune responses via an activation of the inositol requiring enzyme 1α (IRE1 α)/retinoic acid inducible gene-I (RIG1) pathway in tumor cells in mice^9^.

Dietary polyunsaturated fatty acids (PUFAs) are also known to relate to both anticancer effects and carcinogenesis^10^. Supplements containing ω-3 PUFAs may have potential as an effective adjuvant to chemotherapy treatment, and they exert anti-inflammatory effects in cancer patients, including those with advanced lung cancer^11–14^. In contrast, a high dietary intake of ω-6 PUFAs is associated with an increased risk for the development of cancer^15^. Notably, the ratio of serum eicosapentaenoic acid (EPA) to arachidonic acid (AA), or that of serum docosahexaenoic acid (DHA) to AA, may serve as good indicators of the balance between ω-3 PUFAs and ω-6 PUFAs in humans^16^. Moreover, a decreased serum EPA/AA ratio has been reported to be a significant risk factor for cancer-associated death in a Japanese community^17^.

Spencer et al. recently showed that higher dietary fiber consumption affects the gut microbiome and, improves progression-free survival in melanoma patients treated with the immune checkpoint blockade^18^. However, associations between the efficacy of ICI treatment and dietary food consumption before treatment have not been evaluated in lung cancer patients. Therefore, we aimed to evaluate dietary patterns before treatment and examine whether higher dietary seafood consumption and levels of the serum EPA/AA ratio were associated with the overall survival (OS) in NSCLC patients treated with ICI.

## Results

### Patients’ characteristics

Twenty patients with advanced NSCLC who received first-line pembrolizumab treatment were included in this analysis. The baseline characteristics and comparisons between the patients who survived ≥ 3 years after first-line pembrolizumab treatment (long-term survivors [LTS]) and < 3 years after first-line pembrolizumab treatment (short-term survivors [STS]) are presented in Table 1. Twelve (60.0%) and eight (40.0%) patients belonged in the LTS and STS groups, respectively. There were no significant differences in baseline clinical characteristics between the LTS and STS groups.

**Table 1:** Baseline patient characteristics in the short- and long-term survivor groups.

	STS (n = 12)	LTS (n = 8)	*p*-value
OS, median (range), days	504 (185–849)	1234 (1166–1343)	
Age, median (range), years	79.5 (74.0–82.0)	79.0 (70.0–81.0)	0.642
Sex, n (%)			
Male	10 (83.3)	6 (75.0)	0.535
Body mass index, kg/m^2^	22.4 (22.1–23.6)	26.0 (21.6–28.5)	0.757
Obesity, n (%)	2 (16.7)	3 (37.5)	0.296
Smoking status, n (%)			
Current or former smoker	10 (83.3)	8 (100.0)	0.347
Histology type, n (%)			
Squamous	7 (58.3)	5 (62.5)	0.612
Non-squamous	5 (41.7)	3 (37.5)	
Stage, n (%)			
III	2 (16.7)	3 (37.5)	0.535
IV	3 (25.0)	2 (25.0)	
Recurrence	7 (58.3)	3 (37.5)	
PD-L1 TPS, n (%)			
1–49%	1 (8.3)	1 (12.5)	0.653
50–100%	11 (91.7)	7 (87.5)	
Use of lipid-modifying agents, n (%)	3 (25.0)	1 (12.5)	0.465
Use of agents containing EPA, n (%)	0 (0.0)	1 (12.5)	0.400
Serum Alb, median (range), g/dl	3.5 (3.1–4.2)	3.8 (3.5–4.1)	0.588
Serum CRP, median (range), mg/dl	0.7 (0.2–4.3)	1.3 (0.7–2.4)	0.877

Values are presented as medians (interquartile ranges) or frequencies.

*STS* short-term survivor, *LTS* long-term survivor, *OS* overall survival, *BMI* body mass index, *BI* Brinkman index, *PD-L1* programmed cell death ligand 1, *Alb* albumin, *CRP* C-reactive protein.

### Dietary assessment of food consumption

Next, we examined differences in dietary nutrient intakes before ICI treatment between the LTS and STS groups. No significant differences were observed between the two groups in terms of energy, protein, fat, carbohydrate, fiber, and fatty acid intake.

However, in terms of food group intake, the LTS group showed significantly lower intake of sugar and sweeteners than the STS group (*p* = 0.031). In addition, the LTS group had a significantly higher seafood intake than the STS group (*p* = 0.045) (Table 2).

**Table 2:** Daily nutrient and food group intakes in the short- and long-term survivor groups.

	Unit	STS (n = 12)	LTS (n = 8)	*p*-value
Energy/nutrient				
Energy	kcal/d	1497 (1395–2095)	1755 (1306–2064)	0.589
Protein	g/d	52.7 (49.5–65.6)	61.7 (58.9–68.9)	0.877
Fat	g/d	45.8 (40.3–52.2)	52.8 (47.7–59.1)	0.817
Carbohydrate	g/d	197.4 (159.3–226.6)	242.5 (153.1–286.9)	0.939
Total dietary fiber	g/d	8.4 (7.4–14.1)	11.4 (8.7–13.2)	0.537
SFA	g/d	11.3 (10.5–12.5)	14.2 (10.8–15.0)	0.487
MUFA	g/d	18.0 (14.6–19.6)	18.9 (16.9–21.5)	0.589
PUFA	g/d	12.2 (7.8–14.5)	13.9 (10.6–14.3)	0.877
Cholesterol	mg/d	331.0 (230.3–382.8)	291.0 (260.3–304.7)	0.165
Food groups				
Cereals	g/d	332.8 (203.3–477.4)	389.4 (240.2–504.9)	0.758
Potatoes	g/d	13.9 (10.8–46.2)	24.7 (10.3–52.0)	0.908
Sugar and sweeteners	g/d	6.7 (3.8–9.9)	2.4 (0.6–7.7)	**0.031**
Soybean and soybean products	g/d	37.2 (27.9–48.2)	50.2 (40.3–92.5)	0.877
Green and yellow vegetables	g/d	93.0 (44.9–122.9)	95.0 (92.6–110.7)	0.758
Other vegetables	g/d	118.7 (82.8–170.1)	155.6 (60.1–179.2)	0.700
Fruits	g/d	137.3 (31.7–156.6)	145.0 (6.0–193.0)	0.877
Seafoods	g/d	55.7 (24.8–66.6)	70.8 (68.9–74.0)	**0.045**
Meat	g/d	82.0 (50.9–84.5)	75.4 (57.3–83.7)	0.463
Eggs	g/d	48.2 (12.0–59.9	29.1 (27.2–29.9)	0.279
Milk	g/d	121.6 (25.1–155.9)	96.0 (25.7–160.9)	0.418
Oil and fats	g/d	8.8 (6.6–13.4)	9.6 (7.5–11.8)	0.758
Confectionery	g/d	21.0 (0.0–34.3)	8.2 (0.0–13.6)	0.557
Favorite beverages	g/d	598.6 (510.3–1126.0)	675.0 (429.0–768.8)	0.123
Seasonings and spices	g/d	154.6 (110.7–334.7)	294.4 (237.3–329.1)	0.396

Values are presented as medians (interquartile ranges).

*STS* short-term survivor, *LTS* long-term survivor, *SFA* saturated fatty acid, *MUFA* monounsaturated fatty acid, *PUFA* polyunsaturated fatty acid.

### Serum fatty acid analysis

In the serum fatty acid fractionation, the LTS group showed significantly higher levels of serum EPA, an ω-3 PUFA, than the STS group (*p* = 0.023). Additionally, the ratios of serum EPA/AA and DHA/AA were significantly higher in those the LTS group than in the STS group (*p* = 0.009 and 0.021, respectively) (Table 3).

**Table 3:** Serum fatty acids in the short- and long-term survivor groups.

	STS (n = 12)	LTS (n = 8)	*p*-value
AA (μg/ml)	163.0 (143.0–181.0)	142.0 (136.0–170.0)	0.262
EPA (μg/ml)	32.0 (23.0–42.0)	44.0 (33.0–56.0)	**0.023**
DHA (μg/ml)	84.0 (68.0–105.0)	102.5 (84.0–107.0)	0.070
EPA/AA	0.21 (0.14–0.27)	0.33 (0.26–0.38)	**0.009**
DHA/AA	0.59 (0.39–0.65)	0.70 (0.62–0.73)	**0.021**

Values are presented as medians (interquartile ranges).

Significant values are in bold.

*STS* short-term survivor, *LTS, OS* long-term survivor, *AA* arachidonic acid, *EPA* eicosapentaenoic acid, *DHA* docosahexaenoic acid.

Next, we examined the association between the intake of seafood before treatment and the value of each serum fatty acid fraction. A significant negative correlation was found between seafood intake and serum AA values (*r* = − 0.518, *p* = 0.019), and a significant positive correlation was found between seafood intake and the serum EPA/AA ratio (*r* = 0.471, *p* = 0.036) and DHA/AA ratio (*r* = 0.480,* p* = 0.032) (Table 4).

**Table 4:** Correlation between serum fatty acids and seafood intake.

	Spearman’s correlation coefficient	*p*-value
AA	− 0.518	**0.019**
EPA	0.372	0.107
DHA	0.318	0.381
EPA/AA	0.471	**0.036**
DHA/AA	0.480	**0.032**

Significant values are in bold.

*AA* arachidonic acid, *EPA* eicosapentaenoic acid, *DHA* docosahexaenoic acid.

### Association between serum fatty acids and survival

**Figure 1:**
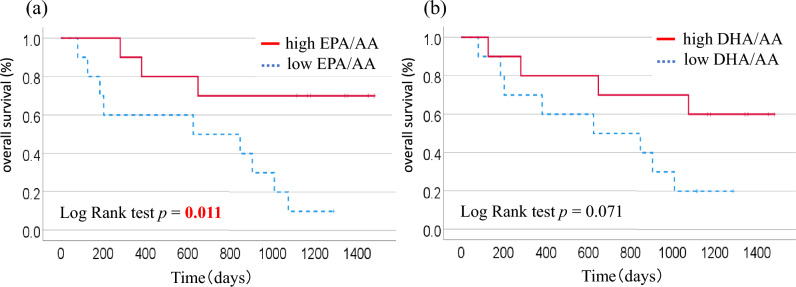
Kaplan–Meier curves of overall survival based on (**a**) serum eicosapentaenoic acid/arachidonic acid (AA) ratio and (**b**) serum docosahexaenoic acid/AA ratio.

Next, we examined whether serum EPA/AA and DHA/AA ratios affected the OS. The median serum EPA/AA ratio was used as the criterion for comparing the OS between the low EPA/AA ratio (< 0.26) (n = 10) and high EPA/AA ratio (≥ 0.26) (n = 10) groups. OS was significantly more prolonged in the high EPA/AA group than the low EPA/AA group (*p* = 0.011) (Fig. 1a). However, no significant difference was found in the OS between the low DHA/AA (< 0.63) (n = 10) and high DHA/AA (≥ 0.63) (n = 10) groups based on the median serum DHA/AA ratio (*p* = 0.071) (Fig. 1b). In univariate analysis, factors significantly associated with OS included EPA/AA ratio, seafoods intakes and smoking status (Supplementary Table S1). In multivariate analysis after adjusting for the major prognostic factors (age, BMI, and smoking status), the EPA/AA ratio remained an independent risk factor for reduced OS (6.72 [95% CI, 1.54–29.38], p = 0.011) (Supplementary Table S2).

## Discussion

This study found that among patients with NSCLC who received initial ICI monotherapy, the LTS group (OS ≥ 3 years) had a higher intake of seafood and higher serum EPA, EPA/AA ratio, and DHA/AA ratio than the STS group (OS < 3 years) before treatment. Moreover the group with a higher serum EPA/AA ratio had significantly more prolonged OS than the group with a lower serum EPA/AA ratio.

Dietary intake of ω-3 PUFAs has been reported to suppress carcinogenesis by inhibiting the inflammatory process, metastasis and tumor proliferation^19^. However, several studies have shown that high intake of ω-6 PUFAs induces the progression of cancer development^20,21^. Thus, the dietary balance between ω-3 and ω-6 PUFAs may be important in determining the roles of PUFAs in carcinogenesis.

Supplementation with fatty acids, especially ω-3 PUFAs, has potentially beneficial effects on immune responses and the maintenance of body weight and skeletal muscle mass in patients with lung cancer^22,23^. ω-3 PUFAs suppress inflammatory responses and enhance antitumor effects^24,25^. For example, they reduce blood levels of inflammatory indicators in patients with cancer undergoing radiotherapy. In a mouse model of obesity associated with breast cancer, administration of fish oil in addition to a high-fat diet decreased the levels of inflammatory cytokines tumor necrosis factor-α and interleukin (IL)-6 and increased the levels of the anti-inflammatory cytokine IL-10^26^. In contrast, ω-6 PUFAs promote inflammation when consumed in excess; conversely, in some studies they have been shown to suppress inflammation. A consistent view regarding this has not been reached^27^. These results suggest that a diet rich in ω-3 PUFAs enhances the efficacy of ICI treatment. Recent clinical studies have reported an association between the therapeutic effect of ICI treatment and blood cholesterol and fatty acid levels in patients with cancer after chemotherapy treatment^28^.

The efficacy of immunotherapy largely depends on the tumor microenvironment (TME). The infiltration of regulatory T cells, myeloid-derived suppressor cells (MDSCs), and tumor-associated macrophages (TAMs) into the TME, or under hypoxic conditions, suppresses the immune function and reduces the efficacy of immunotherapy^29,30^. The ratio of ω-3 to ω-6 PUFA, regulates TAM infiltration and tumor initiation and progression, impacting overall survival (OS)^31^. For example, in breast cancer-transplanted mice with fed cocoa butter, which is high in saturated fatty acids, the differentiation of TAMs is promoted, and the ω-6 PUFAs (AA) induce chronic inflammation through the production of various prostaglandins, promote the accumulation of MDSCs, and suppress immune function around the cancer cells^32^.

Although eicosanoids derived from AA may inhibit inflammation and enhance T cell activity^33,34^, AA also enhances cancer growth by promoting cell proliferation and angiogenesis and inhibiting apoptosis^35,36^. In contrast, the ω-3 PUFA DHA suppresses the expression of hypoxia-induced factor 1α, which is related to cancer growth, in breast cancer cells and it may also inhibit cancer growth by inducing apoptosis in cancer cells^37,38^. Fish oil also inhibits the immunosuppressive effects of saturated fatty acids via chronic inflammation in the TME^39^. In addition, diets rich in ω-3 PUFAs suppress the negative effects of TAMs, thus reducing inflammation and improving immune function; this has been shown in relation to colon and prostate cancer in cachexic mice, which are models of low nutrition^40,41^. Therefore, ω-3 PUFAs may compete with ω-6 PUFAs AA to suppress chronic inflammation and improve immune function in the TME, thereby enhancing the efficacy of immunotherapy^42^. Based on these results, it is inferred that to enhance the beneficial effects of ω-3 PUFAs in fish oil on immune function in patients with cancer, it is necessary to maintain adequate intake of the ω-6 PUFAs AA.

This study found that the high serum EPA/AA ratio group had a significantly more prolonged OS after ICI treatment compared with the low serum EPA/AA ratio group. Nagata et al. has previously shown that decreased levels of serum EPA/AA ratio were associated with higher cancer mortality^17^. However, these associations were not observed in cancer patients but in the general Japanese population. Thus, to the best of our knowledge, this is the first report on the association between treatment efficacy and levels of the serum EPA/AA ratio in cancer patients treated with ICI. In addition, we have also found that higher dietary seafood consumption may improve the OS in ICI-treated cancer patients. Although more than half of the patients treated with ICI therapy fail, we believe that the serum EPA/AA ratio may be a new biomarker that can be used to evaluate the efficacy of ICI therapy and that treatment to increase the serum EPA/AA ratio prior to ICI therapy could enhance the therapeutic effect of ICI therapy.

In the present study, the short-term survival group had a significantly higher intake of sugar and sweeteners than the long-term survival group. Several cancer cells actively take up glucose in an insulin-independent manner, and the glucose taken up is not oxidatively phosphorylated in the mitochondria, which is known as the Warburg effect, but is produced by the glycolytic system for adenosine triphosphate (ATP) production^43,44^. This has been shown to produce ATP faster, and increased lactate production may contribute to evasion of the immune system and metastasis in the cancer microenvironment^45^. Epidemiological studies have reported that sugar and fructose intake increase cancer risk. For example, a prospective cohort study of patients with colon cancer reported an increased risk of recurrence or death in patients who consumed more than two servings/day of sugary beverages compared with those who consumed less than two servings/day^46^. However, more studies are necessary to address the relation between sugar intake and ICI treatment in patients with NSCLC.

This study had several limitations. First, it was a pilot study with a small sample size, resulting in low statistical power. Consequently, it was not possible to infer a causal relationship between the serum EPA/AA ratio and OS in patients undergoing ICI therapy. The effect size necessary for determining the sample size in future large-scale studies was revealed to be 0.3 based on the results of this study. Using a two-tailed, paired t-test with an effect size of 0.30, an α level of 0.05, and a statistical power (1 − β) of 0.80, the required sample size for each group was determined to be 48 individuals. Therefore, further research employing large-scale case studies is necessary to elucidate the relationship between the serum EPA/AA ratio and OS. Second, the assessment of daily nutrient and food group intakes and the measurement of serum PUFA levels were performed once before the treatment. Thus, more follow-up data should be needed to verify data reliability. Third, the measurement of serum PUFA levels were performed only one time before beginning the treatment of ICI. Thus, the data may reflect only recent dietary consumption. Finally, data on the proinflammatory cytokines produced by tumors in our patients were not available. Hence, additional analysis with the proinflammatory cytokines produced by tumors is required.

In conclusion, we showed that higher dietary seafood consumption and higher levels of serum EPA/AA ratio may be associated with longer OS in NSCLC patients treated with ICI. We suggest that level of serum EPA/AA ratio may be a new biomarker for predicting treatment response to ICI in patients with NSCLC and that nutritional interventions that increase the serum EPA/AA ratio could improve survival in cancer patients treated with ICI. Future large-scale studies are required to confirm these findings.

## Materials and methods

### Study design and patient eligibility

This pilot study was conducted between September 1, 2018, and October 31, 2022. Twenty-two patients with NSCLC were included in the study. These patients received ICI monotherapy with pembrolizumab from September 2018 to August 2019 at the National Hospital Organization Osaka Toneyama Medical Center. One patient could not complete the pre-treatment dietary survey, and another refused to participate during the follow-up period. These two patients were therefore excluded, leaving 20 patients for the analysis. This study was conducted in accordance with the 1964 Declaration of Helsinki and its amendments. The purpose of this study was explained to the participants, and written consent was obtained from all participants after sufficient explanation that in the analysis of the survey results, the Privacy Protection Law would be observed, that individuals would be anonymized before analysis as a group, and that withdrawal of consent was possible at any time. This study was approved by the Institutional Review Board of the National Hospital Organization of Osaka Toneyama Medical Center (study number: TNH-2018024). This pilot study aimed to assemble a cohort of 25 cases based on the administration records from the previous year at our hospital. The final sample size was determined by the number of patients treated with pembrolizumab as an initial therapy.

### Dietary survey

A brief-type self-administered diet history questionnaire (BDHQ) was used for the dietary survey; one dietitian interviewed the patients regarding their eating habits in the month before the first ICI administration. Data calculations were outsourced to the DHQ Support Center (Gender Medical Research, Co., Ltd., Tokyo, Japan); dietary data were analyzed. The BDHQ is a four-pages questionnaire with an average response time of 15 min. It is possible to calculate the intake of approximately 30 nutrients and 58 foods using a dedicated nutrient calculator. The validity of the BDHQ has been previously verified^47,48^

### Data collection

Height, weight, age, sex, smoking history, histological type of lung cancer, treatment history, PD-L1 expression rate in tumor tissue, and blood laboratory data before ICI treatment were collected from electronic medical records.

Serum fatty acid fractions were collected before the first ICI administration. A analyses were performed using LC–MS/MS method at a laboratory (LSI Medience Corporation, Tokyo, Japan) with certifications such as ISO 15189, and accreditation by the College of American Pathologists (CAP).

### Survival analysis

OS was defined as the period from the date of the first ICI administration to the end of the follow-up period or death. The STS group comprised patients with an OS of < 3 years, whereas the LTS group comprised patients with an OS of ≥ 3 years. The end of the follow-up period was October 31, 2022 (median follow-up, 1329 days).

### Statistical analyses

The nonparametric Mann–Whitney U test was used to compare the STS and LTS groups in terms of clinical indicators, blood laboratory data, serum fatty acid fractions, and nutrient and food group intake. Fisher’s exact test was used to determine the ratio of patient backgrounds between the two groups. Spearman’s rank correlation coefficient was used to test the association between seafood intake and serum fatty acid fraction. The Kaplan–Meier method was used to calculate OS, and the log-rank test was used for between-group comparison. Cox proportional hazards regression models were employed to assess each factor in both univariate and multivariate analyses. The significance level was set at 5% (two-tailed). IBM SPSS Statistics version 27 was used for the statistical analyses.

### Supplementary Information

Supplementary Tables.

## Data Availability

All data generated or analyzed in this study are included in this published article.

## References

[1] Garcia-Diaz A (2017). Interferon receptor signaling pathways regulating PD-L1 and PD-L2 expression. Cell Rep..

[2] Schadendorf D (2015). Pooled analysis of long-term survival data from phase II and phase III trials of ipilimumab in unresectable or metastatic melanoma. J. Clin. Oncol..

[3] Robert C (2015). Nivolumab in previously untreated melanoma without BRAF mutation. N. Engl. J. Med..

[4] Reck M (2021). Five-year outcomes with pembrolizumab versus chemotherapy for metastatic non-small-cell lung cancer with PD-L1 tumor proportion score ≥ 50. J. Clin. Oncol..

[5] Reck M (2016). Pembrolizumab versus chemotherapy for PD-L1-positive non-small-cell lung cancer. N. Engl. J. Med..

[6] Kanarek N, Petrova B, Sabatini DM (2020). Dietary modifications for enhanced cancer therapy. Nature.

[7] Cuyàs E (2020). Tumor cell-intrinsic immunometabolism and precision nutrition in cancer immunotherapy. Cancers.

[8] Lussier DM, Woolf EC, Johnson JL, Brooks KS, Blattman JN, Scheck AC (2016). Enhanced immunity in a mouse model of malignant glioma is mediated by a therapeutic ketogenic diet. BMC Cancer.

[9] Rubio-Patiño C (2018). Low-protein diet induces IRE1-dependent anticancer immunosurveillance. Cell Metab..

[10] Abel S, Riedel S, Gelderblom W (2014). Dietary PUFA and cancer. Proc. Nutr. Soc..

[11] Finocchiaro C (2012). Effect of n-3 fatty acids on patients with advanced lung cancer: A double-blind, placebo-controlled study. Br. J. Nutr..

[12] Vaughan VC, Hassing MR, Lewandowski PA (2013). Marine polyunsaturated fatty acids and cancer therapy. Br. J. Cancer.

[13] Yang P (2014). Anticancer activity of fish oils against human lung cancer is associated with changes in formation of PGE2 and PGE3 and alteration of Akt phosphorylation. Mol. Carcinog..

[14] D'Eliseo D, Velotti F (2016). Omega-3 fatty acids and cancer cell cytotoxicity: Implications for multi-targeted cancer therapy. J. Clin. Med. Res..

[15] Lawrence GD (2013). Dietary fats and health: Dietary recommendations in the context of scientific evidence. Adv. Nutr..

[16] Yanagisawa N (2010). Polyunsaturated fatty acid levels of serum and red blood cells in apparently healthy Japanese subjects living in an urban area. J. Atheroscler. Thromb..

[17] Nagata M (2017). The ratio of serum eicosapentaenoic acid to arachidonic acid and risk of cancer death in a Japanese community: The Hisayama Study. J. Epidemiol..

[18] Spencer CN (2021). Dietary fiber and probiotics influence the gut microbiome and melanoma immunotherapy response. Science.

[19] Liput KP (2021). Effects of dietary n–3 and n–6 polyunsaturated fatty acids in inflammation and cancerogenesis. Int. J. Mol. Sci..

[20] Zanoaga O (2018). Implications of dietary ω-3 and ω-6 polyunsaturated fatty acids in breast cancer. Exp. Ther. Med..

[21] Huerta-Yépez S, Tirado-Rodriguez AB, Hankinson O (2016). Role of diets rich in omega-3 and omega-6 in the development of cancer. Bol. Med. Hosp. Infant Mex..

[22] van der Meij BS (2010). Oral nutritional supplements containing (n-3) polyunsaturated fatty acids affect the nutritional status of patients with stage III non-small cell lung cancer during multimodality treatment. J. Nutr..

[23] Faber J (2013). Rapid EPA and DHA incorporation and reduced PGE2 levels after one week intervention with a medical food in cancer patients receiving radiotherapy, a randomized trial. Clin. Nutr..

[24] Hao W (2010). Omega-3 fatty acids suppress inflammatory cytokine production by macrophages and hepatocytes. J. Pediatr. Surg..

[25] Das UN (2019). Can bioactive lipids augment anti-cancer action of immunotherapy and prevent cytokine storm?. Arch. Med. Res..

[26] Innes JK, Calder PC (2018). Omega-6 fatty acids and inflammation. Prostagl. Leukot. Essent. Fatty Acids.

[27] Monk JM (2021). Fish oil supplementation increases expression of mammary tumor apoptosis mediators and reduces inflammation in an obesity-associated HER-2 breast cancer model. J. Nutr. Biochem..

[28] Karayama M (2022). Increased serum cholesterol and long-chain fatty acid levels are associated with the efficacy of nivolumab in patients with non-small cell lung cancer. Cancer Immunol. Immunother..

[29] Wang B (2021). Targeting hypoxia in the tumor microenvironment: A potential strategy to improve cancer immunotherapy. J. Exp. Clin. Cancer Res..

[30] Wang S, Xie K, Liu T (2021). Cancer immunotherapies: From efficacy to resistance mechanisms: Not only checkpoint matters. Front. Immunol..

[31] Khadge S, Sharp JG, Thiele GM, McGuire TR, Talmadge JE (2020). Fatty acid mediators in the tumor microenvironment. Adv. Exp. Med. Biol..

[32] Sinha P, Clements VK, Fulton AM, Ostrand-Rosenberg S (2007). Prostaglandin E2 promotes tumor progression by inducing myeloid-derived suppressor cells. Cancer Res..

[33] Serhan CN (2005). Lipoxins and aspirin-triggered 15-epi-lipoxins are the first lipid mediators of endogenous anti-inflammation and resolution. Prostagl. Leukot. Essent. Fat. Acids.

[34] Kumar J, Gurav R, Kale V, Limaye L (2014). Exogenous addition of arachidonic acid to the culture media enhances the functionality of dendritic cells for their possible use in cancer immunotherapy. PLoS ONE.

[35] Leahy KM (2002). Cyclooxygenase-2 inhibition by celecoxib reduces proliferation and induces apoptosis in angiogenic endothelial cells in vivo. Cancer Res..

[36] Cianchi F (2001). Up-regulation of cyclooxygenase-2 gene expression correlates with tumor angiogenesis in human colorectal cancer. Gastroenterology.

[37] Mouradian M (2015). Docosahexaenoic acid attenuates breast cancer cell metabolism and the Warburg phenotype by targeting bioenergetic function. Mol. Carcinog..

[38] Skender B (2014). DHA-mediated enhancement of TRAIL-induced apoptosis in colon cancer cells is associated with engagement of mitochondria and specific alterations in sphingolipid metabolism. Biochim. Biophys. Acta.

[39] Liu L (2020). Consumption of the fish oil high fat diet uncouples obesity and mammary tumor growth through induction of reactive oxygen species in protumor macrophages. Cancer Res..

[40] Faber J (2008). Beneficial immune modulatory effects of a specific nutritional combination in a murine model for cancer cachexia. Br. J. Cancer.

[41] Liang P (2020). Effect of dietary omega-3 fatty acids on castrate-resistant prostate cancer and tumor-associated macrophages. Prostate Cancer Prostatic Dis..

[42] Wall R, Ross RP, Fitzgerald GF, Stanton C (2010). Fatty acids from fish: Anti-inflammatory potential of long-chain omega-3 fatty acids. Nutr. Rev..

[43] Koppenol WH, Bounds PL, Dang CV (2011). Otto Warburg’s contributions to current concepts of cancer metabolism. Nat. Rev. Cancer.

[44] Hsu PP, Sabatini DM (2008). Cancer cell metabolism: Warburg and beyond. Cell.

[45] Cairns RA, Harris IS, Mak TW (2011). Regulation of cancer cell metabolism. Nat. Rev. Cancer.

[46] Fuchs MA (2014). Sugar-sweetened beverage intake and cancer recurrence and survival in CALGB 89803 (Alliance). PLoS ONE.

[47] Kobayashi S (2011). Comparison of relative validity of food group intakes estimated by comprehensive and brief-type self-administered diet history questionnaires against 16 d dietary records in Japanese adults. Public Health Nutr..

[48] Kobayashi S (2012). Both comprehensive and brief self-administered diet history questionnaires satisfactorily rank nutrient intakes in Japanese adults. J. Epidemiol..

