# Supplementary material for: Impact of serum eicosapentaenoic acid/arachidonic acid ratio on overall survival in lung cancer patients treated with pembrolizumab: a pilot study
Source: Sci Rep. 2024 Jan 16;14:1384. doi: 10.1038/s41598-024-51967-y (PMC10792072; doi:10.1038/s41598-024-51967-y)
Supplement: Supplementary file 1 — Supplementary Tables. [file 41598_2024_51967_MOESM1_ESM.pdf]

Impact of serum eicosapentaenoic acid/arachidonic acid ratio on overall survival in lung cancer patients treated with pembrolizumab: A pilot study

Ikue Tanaka[1], Yukihiro Yano[2], Masahide Mori[2], Satoru Manabe[4] &, Keisuke Fukuo[1,3]*

[1]Department of Food Sciences and Nutrition Major, Graduate School of Human Environmental Science, Mukogawa Women's University
[2]Department of Thoracic Oncology, National Hospital Organization, NHO Osaka Toneyama Medical Center
[3]Research Institute for Nutrition Sciences, Mukogawa Women's University
[4]Department of Nutrition, National Hospital Organization, NHO Osaka Toneyama Medical Center

*Corresponding author
Keisuke Fukuo
Professor and director of Research Institute for Nutrition Sciences, Mukogawa Women's University
6-46 Ikebiraki-Cho, Nishinomiya, Hyogo, 663-8558, Japan.
Phone number: +81-798-45-9922, Email address: fukuo@mukogawa-u.ac.jp

Supplementary Table S1. Factors associated with overall survival (OS) in univariate analysis.

	HR (95%CI)	p-value
Age		
≥ 79	Reference	
< 79	0.84 (0.27 - 2.63)	0.771
Sex		
Female	Reference	
Male	1.14 (0.25 - 5.21)	0.866
BMI		
≥ 22.4	Reference	
< 22.4	1.18 (0.38 - 3.68)	0.778
Smoking status		
Never	Reference	
Curent or former smoker	0.18 (0.03 - 0.92)	**0.039**
Histology type		
Squamous	Reference	
Non-Squamous	0.99 (0.31 - 3.13)	0.985
PD-L1 TPS		
90-100%	Reference	
50-89%	3.15 (0.68 - 14.69)	0.143
1-49%	1.59 (0.14 - 17.66)	0.704
Serum Alb		
≥ 3.65	Reference	
< 3.65	1.67 (0.53 - 5.28)	0.382
Serum CRP		
≥ 0.96	Reference	
< 0.96	0.77 (0.25 - 2.41)	0.657
Sugar and Sweeteners intakes		
≥ 4.04	Reference	
< 4.04	0.44 (0.13 - 1.47)	0.181
Seafoods intakes		
≥ 63.16	Reference	
< 63.16	3.67 (1.08 - 12.48)	**0.037**
EPA/AA		
≥ 0.26	Reference	
< 0.26	4.80 (1.28 - 18.00)	**0.020**
DHA/AA		
≥ 0.63	Reference	
< 0.63	2.93 (0.87 - 9.91)	**0.084**

HR, hazard ratio; CI, confidence interval; BMI, Body Mass Index; BI,Brinkman index; PD-L1,Programmed cell death-ligand 1; Alb,albumin; CRP,C-reactive protein; AA,Arachidonic acid; EPA,eicosapentaenoic acid; DHA,docosahexaenoic acid

Supplementary Table S2. Factors associated with overall survival (OS) in multivariate analysis.

	HR (95% CI)	p-value
Age		
≥ 79	Reference	
< 79	0.60 (0.15 - 2.45)	0.480
BMI		
≥ 22.4	Reference	
< 22.4	1.74 (0.43 - 7.01)	0.433
Smoking status		
Never	Reference	
Curent or former smoker	0.66 (0.08 - 5.40)	0.701
EPA/AA		
≥ 0.26	Reference	
< 0.26	6.48 (1.26 - 33.37)	**0.026**

HR, hazard ratio; CI, confidence interval; BMI, Body Mass Index; BI,Brinkman index; PD-L1,Programmed cell death-ligand 1; Alb,albumin; CRP,C-reactive protein; AA,Arachidonic acid; EPA,eicosapentaenoic acid; DHA,docosahexaenoic acid
